# A Study of Time to Recovery Following Loss of Neuromonitoring Signal of the Recurrent Laryngeal Nerve in Thyroid Surgery

**DOI:** 10.1002/wjs.70254

**Published:** 2026-02-16

**Authors:** Joyce Yu, Rathina Ragavan, Carolina Nylen, Ahmad Aniss, Stan Sidhu, Alexander Papachristos, Daniel Novakovic, Thomas Stewart, Karl Ng, Hakan Ozoran, Mark Sywak

**Affiliations:** ^1^ University of Sydney Camperdown NSW Australia; ^2^ Macquarie University Macquarie Park NSW Australia; ^3^ Department of Molecular Medicine and Surgery Karolinska Institutet Stockholm Sweden; ^4^ University of Sydney Endocrine Surgical Unit St Leonards Australia; ^5^ University of Sydney Voice Lab Camperdown NSW Australia; ^6^ Department of Neurology and Clinical Neurophysiology Royal North Shore Hospital Sydney Australia; ^7^ Clinical Medical School University of Oxford Oxford UK

**Keywords:** loss of signal, neuromonitoring, recurrent laryngeal nerve injury, thyroidectomy

## Abstract

**Background:**

Injuries of the recurrent laryngeal nerve (RLN) during thyroidectomy although infrequent can lead to major morbidity. Permanent RLN injury is uncommon; however, temporary neurapraxia and loss of signal (LOS) during intraoperative neuromonitoring (IONM) are seen more frequently. This study aimed to identify factors associated with type I (segmental) and II (global) LOS of the RLN during thyroid surgery and to analyze time to recovery of vocal cord function.

**Methods:**

This observational retrospective cohort study included 3806 patients (2924 female, 76.8% and 882 male, 23.2%) who underwent hemi or total thyroidectomy in a tertiary center between January 2015 and March 2021. Regression analyses determined factors associated with LOS. Postoperative fibreoptic laryngoscopy was used to measure time to recovery of vocal cord function.

**Results:**

RLN LOS occurred in 167 (2.7%) of 5983 nerves at risk during surgery. The rate of Type I and Type II LOS per nerve at risk was 1.4% and 1.3%, respectively. Compared with an indication of malignancy, toxic nodule was associated with 96% increased odds of LOS independent of age and sex (*P* < 0.001). Time to recovery was reduced for those with a Type II LOS (median 4 weeks) compared to Type I LOS (median 8 weeks and *p* = 0.04). Female sex and increasing age were independently associated with a longer duration to return of vocal cord function.

**Conclusions:**

Time to recovery of RLN function is significantly reduced for patients with Type II LOS. Toxic thyroid nodules were associated with a higher risk of LOS, and female sex and age are significantly associated with a longer time to recovery.

## 1 Introduction

Recurrent laryngeal nerve (RLN) injury is an uncommon but significant complication in thyroid surgery. Unilateral injury can lead to dysphonia and reduced quality of life, whereas bilateral injury can lead to life threatening respiratory distress and the potential need for tracheostomy [1]. In recent literature, the rate of transient RLN injury following thyroidectomy was 2%–11% and permanent injury was seen in 0.6%–1.6% [2, 3]. Since it was first proposed by Lahey in 1938, the accepted ‘gold standard’ to reduce the risk of injury has been routine visual identification of the RLN [4, 5]. However, RLN neurapraxia may still occur with a structurally intact nerve and only 10%–15% of RLN palsies may be recognized at surgery [6]. Consequently, intraoperative nerve monitoring (IONM) has gained popularity as a valuable adjunct which can provide real‐time feedback regarding nerve function and aid intraoperative decision making. A widely accepted benefit of IONM is that it has excellent negative predictive value (99.7%) meaning that a positive signal after hemithyroidectomy confirms it is safe to complete the contralateral thyroidectomy in cases where total thyroidectomy is planned [7]. If LOS is recognized, the surgeon will generally consider a staged procedure intraoperatively, delaying the contralateral hemithyroidectomy until improvement in vocal cord function has been confirmed [8, 9, 10].

Although IONM is being increasingly utilized worldwide, evidence of its efficacy in preventing RLN injury remains inconclusive. Although a recent meta‐analysis showed that IONM is associated with a reduced incidence of both transient and permanent RLN injury after thyroidectomy, other meta‐analyses have found there is no statistically significant reduction compared to direct visualization alone [11, 12, 13, 14]. Studies have reported benefit only in high risk scenarios [15]. Some more recent population‐based studies are more promising; with a UK study including 40,000 thyroidectomies demonstrated significant reduction in both transient and permanent RLN palsy, and a US study including 6942 thyroidectomies demonstrated significant reduction for severe RLN injury [2, 3].

There is a paucity of data regarding the predictive factors for LOS, and the relationship between type of LOS and recovery time. Therefore, this study aims to identify predictive factors for LOS in thyroid surgery and to make observations regarding the time to recovery when LOS has occurred.

## 2 Material and Methods

### 2.1 Study Design

An observational retrospective cohort study of all patients who underwent hemi or total thyroidectomy in a tertiary center between January 2015 and March 2021 was undertaken. Exclusion criteria included: age at operation < 18 years, the presence of preexisting vocal cord palsy, or if the RLN was sacrificed due to malignant invasion.

Patient data were retrieved from the hospital‐based endocrine surgery quality register which includes a record of all endocrine surgery procedures performed within the unit. These data are de‐identified and stored in a hospital based server following each procedure and follow‐up event. Ethics approval for this study was provided by the Northern Sydney Local Health District Human Research Ethics Committee (Reference Number: 2019/ETH13065).

### 2.2 Parameters and Outcomes

The primary outcome measure was LOS, which is defined as complete signal loss at the conclusion of the procedure during IONM of the RLN. A loss of signal (LOS) is defined as a decrease in amplitude to less than 100 μV (μV) or a complete absence of signal. Type I LOS is defined as segmental injury, characterized by a loss of nerve signal distal to the point of damage but preserved proximally; Type II LOS is defined as global injury, characterized by a complete loss of nerve signal of the vagus and RLN on the side of injury [16]. Time to recovery is presented in number of weeks taken for complete symptomatic recovery of loss of nerve dysfunction.

Demographic variables collected include age at operation and sex. Other variables included indication, grouped into categories of malignancy (including lesions suspicious for and confirmed malignancy), compressive goiter, benign toxic lesion, benign nontoxic lesion, Graves, and Other (e.g., thyroidectomy in conjunction with parathyroidectomy). Type of surgery refers to whether the patient underwent total or hemithyroidectomy. The thyroidectomy procedure type was classified as a primary procedure, secondary, or reoperative procedure, completion thyroidectomy following previous hemithyroidectomy for cancer, or surgery for recurrent cancer. Lymph node dissection was grouped into submental or submandibular (I), lateral (groups II–V), or central (VI–VII). Tumor size was measured in mm and represented the maximal diameter.

### 2.3 Surgical Technique

Nerve integrity monitoring was perfomed routinely for all thyroidectomy procedures in this study using the NIM 3.0 and NIM VITAL Systems (Medtronic, Minneapolis, MN, USA). IONM was perfomed using a TriVantage endotracheal EMG tube which detects vocal cord depolarization upon stimulation of the RLN or vagus nerve, which is visually produced as an electromyogram on a monitor) (Video 1) [6]. During intubation, an anesthetist confirmed correct endotracheal tube positioning under direct vision video laryngoscopy. The minimum recordable amplitude was set at 100 μV, with responses below this threshold considered absent due to signal‐to‐noise limitations. Electrode stimulation was performed at 1 mA. During the operation, a standardized four step procedure was used to test the vagus nerve and RLN: V1 baseline signal obtained from the ipsilateral vagus nerve; R1 signal obtained from RLN upon first identification; R2 signal obtained from RLN after complete dissection from Berry's ligament; and V2 signal obtained from the ipsilateral vagus nerve after hemostasis of the surgical field.

**Video 1:** Intermittent neuromonitoring of the recurrent laryngeal nerve during thyroidectomy using Medtronic NIM Vital. (To view this video in the full‐text HTML version of the article, please visit https://onlinelibrary.wiley.com/doi/10.1002/WJS.70254).

### 2.4 Verification of Vocal Cord Function

In this study, all patients underwent preoperative laryngeal assessment either with fibreoptic laryngoscopy or transcutaneous laryngeal ultrasonography to confirm vocal cord movement. If there was any LOS, the patient underwent fibreoptic laryngoscopy at postoperative day 1 to confirm vocal cord palsy. Patients with confirmed vocal cord palsy were reviewed monthly by an ENT surgeon until recovery, defined as either purposeful vocal cord movement on laryngoscopy or resolution of voice symptoms. Some patients were offered immediate injection laryngoplasty, speech therapy, or nimodipine. Vocal cord palsies lasting > 6 months were considered permanent.

### 2.5 Statistical Analyses

The majority of continuous variables were not normally distributed and are presented as medians (Quartile 1 and Quartile 3). Categorical variables are presented as *n* (%). Comparisons of continuous variables between two groups were made using the Mann–Whitney *U* test, and comparisons of categorical variables were made using Pearson's chi‐squared test.

Logistic and linear regression were used to assess the association between LOS and predictors including procedure type, indication, tumor size, and node dissection. Linear regression analyses were used to determine association between time to recovery and predictors of procedure type, indication, tumor size, and node dissection, adjusted for age and sex. All analyses were performed using Stata 11.2 (StataCorp, College Station, Texas, United States).

## 3 Results

A total of 3806 patients underwent either total (*n* = 2123; 55.8%) or hemithyroidectomy (*n* = 1683; 44.2%). Among these patients, a higher percentage of women underwent thyroid procedures compared to men (2924 female, 76.8% and 882 male, 23.2%) (Table 1). Additionally, women who underwent these procedures were younger than men, with a median age of 55 years old (*p* < 0.001). The most common indications were malignancy (38.6%) and compressive goiter (35.5%). Both men and women had a greater proportion of total thyroidectomies than hemithyroidectomies (*p* < 0.001), with over 90% being a primary procedure (*p* = 0.04).

**TABLE 1:** Details of procedure and loss of signal by patient sex.

Characteristics	Men (*n* = 882)	Women (*n* = 2924)	*p*
Age (years; median Q1,Q3)	60 (48, 70)	55 (42, 66)	**< 0.001**
Procedure details			
Indication			
Malignancy	385 (47.1)	1086 (40.2)	**< 0.001**
Compressive goiter	301 (36.8)	1050 (38.9)	0.3
Benign nontoxic	3 (0.4)	15 (0.6)	0.5
Benign toxic	82 (10.0)	250 (9.3)	0.5
Graves	31 (3.8)	243 (9.0)	**< 0.001**
Other	15 (1.8)	59 (2.2)	0.5
Type of surgery			
Total thyroidectomy	445 (50.5)	1678 (57.4)	**< 0.001**
Hemithyroidectomy	437 (49.6)	1246 (42.6)	**< 0.001**
Procedure type			
Primary	808 (91.6)	2650 (90.7)	0.4
Secondary	25 (2.8)	140 (4.8)	**0.01**
Completion for cancer	39 (4.4)	110 (3.8)	0.3
Further surgery for cancer	10 (1.1)	21 (0.7)	0.2
Lymph node dissection			
Central	122 (13.8)	337 (11.5)	0.3
Lateral	109 (12.4)	283 (9.6)	0.2
Nerves at risk			
One	176 (38.7)	578 (35.4)	0.9
Two	279 (61.3)	1056 (64.4)	**0.01**
Loss of signal			
No loss	722 (94.1)	2477 (95.6)	**0.04**
Type I	17 (2.2)	68 (2.6)	0.4
Type II	28 (3.7)	48 (1.8)	**< 0.001**

*Note:* Continuous variables are presented as median (quartile 1 and quartile 3). Categorical variables are presented as *n* (%). *p*‐values, comparing women with men, were generated using the Mann–Whitney *U* test for continuous variables and the X^2^ test for categorical variables. Bold values indicate *p* < 0.05.

A total of 161 patients had LOS, with a median age of 59 years old (Table 2). There was no difference in the rate of LOS observed between men and women. There were a total of 5983 nerves at risk, and the overall rate of LOS per nerve at risk was 2.7% (*n* = 161). The rate of Type I and Type II LOS per nerve at risk was 1.4% and 1.3%, respectively. There were 3 cases of permanent RLN injury (0.05%). Most patients who had LOS had undergone a thyroid procedure for an indication of malignancy (39.3%) or compression (34.2%; *P* < 0.05; and Table 2). Proportion of lateral node dissections were greater in those with loss of signal (13.0%) compared to those who had no loss of signal (8.4%; *P* = 0.04; and Table 2).

**TABLE 2:** Patient demographics and details of procedure by loss of signal.

Characteristics	No loss (*n* = 3202)	Loss of signal (*n* = 161)	*p*
Age (years; median Q1,Q3)	57 (43, 68)	59 (46, 69)	0.2
Females	2477 (77.4)	116 (72.1)	0.1
Procedure details			
Indication			
Malignancy	1236 (41.1)	57 (39.3)	**0.04**
Compressive goiter	1162 (38.7)	51 (34.2)	0.2
Benign nontoxic	11 (0.4)	0 (0.0)	0.4
Benign toxic	295 (9.8)	27 (18.1)	**< 0.001**
Graves	239 (8.0)	12 (8.1)	0.9
Other	63 (2.1)	2 (1.3)	0.5
Type of surgery			
Total thyroidectomy	1890 (59.0)	68 (42.2)	**< 0.001**
Hemithyroidectomy	1312 (41.0)	93 (57.8)	**< 0.001**
Procedure type			
Primary	2938 (91.8)	147 (91.3)	0.8
Secondary	135 (4.2)	11 (6.3)	0.1
Completion for cancer	122 (3.8)	3 (1.9)	0.2
Further surgery for cancer	6 (0.2)	0 (0.0)	0.5
Lymph node dissection			
Central	375 (11.7)	16 (9.9)	0.5
Lateral	269 (8.4)	21 (13.0)	**0.04**
Nerves at risk			
One	698 (35.1)	57 (56.4)	**< 0.001**
Two	1293 (64.9)	44 (43.6)	

*Note:* Continuous variables are presented as median (quartile 1 and quartile 3). Categorical variables are presented as *n* (%). *p*‐values, comparing women with men, were generated using the Mann–Whitney *U* test for continuous variables and the X^2^ test for categorical variables. Bold values indicate *p* < 0.05.

Regarding factors associated with LOS, an indication for surgery of benign toxic nodule was associated with a 2.1 fold increased risk LOS independent of age and sex compared with an indication of malignancy (*P* = 0.02) (Table 3). We did not find any direct predictable association between LOS and indication for surgery, lymph node dissection or tumor size.

**TABLE 3:** Association between demographics, procedural factors, and likelihood of loss of signal.

Variable	OR (95% CI)	*P*_OR_	AOR (95% CI)	*P*_AOR_
Females	1.33 (0.93–1.89)	0.1	1.27 (0.88–1.80)	0.2
Age	1.00 (0.38–1.00)	0.4	1.00 (0.99–1.01)	0.4
Indication				
Malignancy	1.00		1.00	
Compressive goiter	0.95 (0.64–1.40)	0.8	0.96 (0.59–1.54)	0.8
Benign nontoxic	—		—	
Benign toxic	1.98 (1.23–3.19)	**< 0.01**	2.10 (1.11–3.96)	**0.02**
Graves	1.09 (0.57–2.06)	0.8	2.18 (0.99–4.80)	0.05
Other	0.69 (0.16–2.88)		—	
Type of surgery				
Total thyroidectomy	—		—	
Hemithyroidectomy	1.97 (1.43–2.71)	**< 0.001**	1.95 (1.41–2.68)	**< 0.001**
Lateral node dissection	1.63 (0.87–2.65)	0.1	1.56 (0.88–2.77)	0.1
Tumor size (mm)	1.00 (0.98–1.02)	0.9	0.99 (0.98–1.00)	0.5

*Note:* Data are presented as odds ratios (ORs) with 95% confidence intervals (95% CIs). *p*‐values were generated using univariable and multivariable logistic regression analyses. Adjusted odds ratios (AOR) are adjusted for sex and age. Bold values indicate *p* < 0.05.

**FIGURE 1:**
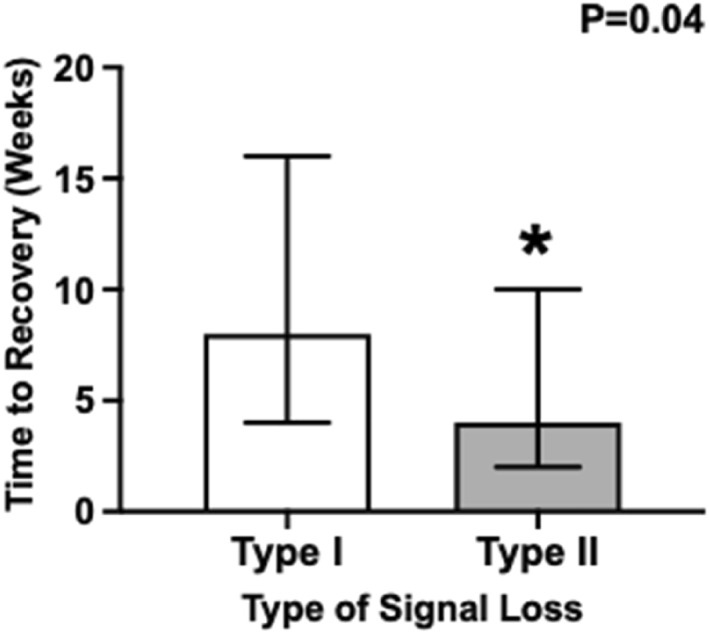
Time to recovery in weeks following Types I and II loss of recurrent laryngeal nerve signal. Data are presented as median (Q1 and Q3) weeks to recovery following loss of signal. *Denotes a difference between Type I and Type II at *P* < 0.05. *p*‐value generated using Wilcoxon rank‐sum test of difference.

The time to recovery of vocal cord function was significantly shorter for those who had a Type II LOS (median 4 weeks) compared to those who had a Type I LOS (median 8 weeks and *p* = 0.04) (Figure 1). In our regression analysis, Type II LOS was associated with 4.62 weeks reduced duration to recovery compared to Type I LOS (Table 4). On the other hand, being female, increasing age and undergoing hemithyroidectomy were each independently associated with a longer duration to recovery after LOS (Table 4).

**TABLE 4:** Association between demographics, procedural factors, and time to recovery.

Variable	B‐coef (95% CI)	*P*_OR_	Adjusted B‐coef (95% CI)	*P*_AIRR_
Females	3.82 (1.21–5.23)	**< 0.05**	3.23 (1.19–5.26)	**< 0.05**
Age	1.09 (1.02–1.17)	**< 0.05**	1.08 (1.01–1.16)	**< 0.05**
Indication				
Malignancy	Ref		Ref	
Compressive goiter	0.15 (−4.93–5.24)	0.9	−0.75 (−6.01–4.5)	0.7
Benign non‐toxic	—		—	
Benign toxic	−0.62 (−6.55–5.30)	0.8	−1.10 (−7.20–4.99)	0.3
Graves	−3.88 (−12.1–4.41)	0.4	−2.59 (−10.9–5.7)	0.5
Other	1.40 (−14.8–17.6)	0.8	3.59 (−12.6–19.8)	0.6
Type of surgery				
Total thyroidectomy	Ref		Ref	
Hemithyroidectomy	2.84 (2.79–2.88)	**< 0.05**	1.84 (1.81–1.87)	**< 0.05**
Node dissection	1.75 (−3.26–6.76)	< 0.001	1.81 (−3.48–7.11)	< 0.001
Tumor size (mm)	−0.05 (−0.14–0.03)	0.2	−0.05 (−0.14–0.03)	0.1
Type I	Ref		Ref	
Type II	−4.12 (−8.02–−0.20)	**0.03**	−4.62 (−8.51–0.72)	**0.02**

*Note:* Data are presented as beta coefficients with 95% confidence intervals (95% CIs). *p*‐values were generated using univariable and multivariable linear regression analyses. Adjusted beta coefficients are adjusted for sex and age. Bold values indicate *p* < 0.05.

## 4 Discussion

This large cohort study describes our experience with LOS of the RLN as shown by intermittent neuromonitoring during thyoid surgery. This study demonstrates that time to recovery is significantly reduced for Type II compared to Type I LOS (median 4 vs. 8 weeks and *p* = 0.04). Factors associated with increased time to recovery include female sex and increasing age. This result is supported by studies from the International Neural Monitoring Study Group, which found that Type I LOS entails more severe nerve damage than Type II and took significantly longer to recover, with similar time median duration (median 27 vs. 62 days and *p* < 0.001) [16, 17]. It also affected women disproportionately. Other studies have also found that Type II LOS recovers more quickly than Type I injury [18]. An explanation for this may be that Type II LOS is related to stretch injury, causing axonal elongation which can be tolerated due to a degree of nerve elasticity, and hence usually results in neurapraxia rather than a permanent injury. This may result in a lesser disruption of axonal function with an associated shorter period of nerve dysfunction. In comparison, Type I LOS where immediate damage can be caused by local thermal injury or direct surgical trauma to the RLN, the axonal injury is likely more extensive, or at least stretched over a shorter distance with less axonal elasticity, with an associated longer time to restoration of nerve function [16]. Although some patients were offered interventions, such as immediate injection laryngoplasty or speech therapy, these were not required in this case series as most patients experienced spontaneous recovery of nerve function. Two patients were trialled on nimodipine for 4 weeks under the guidance of the ENT surgeon. Due to the small number of treated patients and incomplete documentation, we were unable to evaluate effect on time to recovery. Nonetheless, existing literature suggests that calcium channel blockers, such as nimodipine, may enhance recovery following peripheral nerve injury, and this could be a potential area of future investigation [19]. Overall, our results contribute to the evolving body of evidence, which can help to provide information about the expected recovery time of vocal cord movement.

In our multivariate regression model, thyroidectomy for a benign toxic lesion was associated with significantly higher odds of LOS compared to malignant nodules, independent of age and sex (*p* = 0.02). It is unclear why this would be the case and would require further investigation and validation. Importantly, we did not find an association between LOS and other indications such as retrosternal goiter, malignancy, or tumor size.

Although a higher proportion of patients with LOS underwent lateral node dissection, this association was not detected in the multivariable regression model. This demonstrates that although lateral node dissection may co‐occur with LOS, it is not an independent predictor, potentially due to confounding variables or collinearity with other variables. Additionally, we speculate the lack of association may reflect limited mechanical retraction on the RLN during lateral node dissection. This is interesting as it is generally thought that RLN injury is higher in cases of thyroid carcinoma, Graves' disease and large goiter—conditions typically associated with more challenging dissections due to disruption of tissue planes and increased traction [3, 13].

In this study, hemithyroidectomies were associated with 95% increased odds of LOS, independent of age and sex, compared to total thyroidectomies (*p* < 0.001). This most likely reflects the intraoperative use of IONM to guide decision‐making toward a staged thyroidectomy after LOS is encountered on the first side. This introduces selection bias as some hemithyroidectomies may reflect intraoperative decisions rather than planned procedures. It is also possible that patient‐specific factors influence susceptibility to LOS, and that a LOS on the first side may increase the likelihood of its occurrence on the contralateral side. Further investigation is needed to explore this hypothesis, particularly with data that can distinguish between ‘true’ hemithyroidectomies and those initially intended as total thyroidectomies.

## 5 Limitations

This study has several limitations inherent to its retrospective cross‐sectional design, including the potential for reporting bias, selection bias, misclassification, input error, and missing data. Measurement of time to recovery of vocal cord movement relied on monthly postoperative laryngoscopy and patient‐reported voice function which may have reduced its precision. Additionally, to preserve statistical power in the regression model, the initial 12 surgical indications (compression, growth, risk of malignancy, malignancy, single nodule (nontoxic), multinodular goiter (nontoxic), retrosternal goiter, single nodule (toxic), multinodular goiter (toxic), Graves' disease, other, and unknown) were collapsed into broader categories, which may have introduced classification bias. Though nodule size was analyzed between patients with and without LOS, incomplete data limited comparisons across other indications, such as toxic versus nontoxic nodules. Length of surgery was also not recorded but should be considered in future studies as a marker of operative complexity. Furthermore, we were unable to distinguish between true hemithyroidectomies and those initially intended as total thyroidectomies, limiting our ability to investigate the role of patient‐specific factors on the incidence of LOS.

Nonetheless, the strength of our study lies within the novelty in our results compared with other studies in that we have provided a quantifiable estimated difference in time to recovery by sex, increasing age, and type of LOS.

## 6 Conclusion

Overall, this study supports emerging evidence that Type I LOS is more severe compared to Type II LOS and has found that time to recovery of vocal cord function is significantly reduced for patients with Type II LOS. Our observations demonstrate that RLN function on average returns to normal 4 weeks after Type II LOS and 8 weeks after Type I LOS. This may help counsel patients on expected recovery time when RLN injury is experienced. Our data demonstrate that female sex and age are significantly associated with a longer time to recovery.

## Author Contributions

**Joyce Yu:** data curation, formal analysis, investigation, visualization, writing – original draft, writing – review and editing, project administration. **Rathina Ragavan:** formal analysis, data curation, investigation, software, visualization, writing – review and editing. **Carolina Nylen:** conceptualization, methodology. **Ahmad Aniss:** project administration, data curation. **Stan Sidhu:** conceptualization, data curation, methodology, writing – review and editing. **Alexander Papachristos:** conceptualization, methodology, writing – review and editing. **Daniel Novakovic:** data curation, writing – review and editing. **Thomas Stewart:** data curation, writing – review and editing. **Karl Ng:** writing – review and editing. **Hakan Ozoran:** writing – review and editing. **Mark Sywak:** conceptualization, supervision, data curation, methodology, writing – review and editing.

## Funding

The authors have nothing to report.

## Ethics Statement

Provided by the Northern Sydney Local Health District Human Research Ethics Committee (Reference Number: 2019/ETH13065).

## Conflicts of Interest

The authors declare no conflicts of interest.

## Data Availability

The data that support the findings of this study are available from the corresponding author upon request. The data are not publicly available due to privacy or ethical restrictions.
